# To Physicians

**Published:** 1877-08

**Authors:** 


Physicians in want of Surgical Instruments would do well to correspond with the Publisher.

